# Spatial Distribution of Straw‐Colored Fruit Bats (*Eidolon helvum*) Roosts in Obafemi Awolowo University

**DOI:** 10.1002/ece3.72084

**Published:** 2025-08-29

**Authors:** Manuel Ndebele, Mary Ebun Ajibola, Suara AbdulAfeez, Pamilerin Ajumobi, Busayo Aina, Faith Adeniran, Toheeb Oladapo, Jerimiah Chakuya

**Affiliations:** ^1^ Department of Geospatial Intelligence Zimbabwe National Geospatial and Space Agency Harare Zimbabwe; ^2^ African Regional Centre for Space Science and Technology Education – English (ARCSSTEE) Ile‐Ife Nigeria; ^3^ Department of Zoology Obafemi Awolowo University Ile‐Ife Nigeria; ^4^ Scientific Services Zimbabwe Parks and Wildlife Management Authority Harare Zimbabwe

**Keywords:** GIS, Kernel density estimation, Obafemi Awolowo University, roosting sites, straw‐colored fruit bats

## Abstract

Bats are essential to ecosystem functioning, providing vital services such as pollination, seed dispersal, and insect control. With over 1400 species worldwide, they exhibit diverse roosting behaviors that are influenced by both natural and anthropogenic factors. However, research on bat populations, particularly in urban environments, remains limited in Nigeria. The study investigated the spatial distribution of Straw‐Colored Fruit Bat ( *Eidolon helvum* ) roosts within the Obafemi Awolowo University (OAU) campus in Nigeria. Using field surveys and Geographic Information Systems (GIS) analysis, the researchers mapped bat roost distribution, identified hotspots, and explored tree species preferences. Our findings reveal that roosting sites are predominantly clustered in sections of the campus, particularly in the Faculty of Science and Faculty of Administration areas, with 83% of observations recorded in Section D. *Celtis zenkeri* was identified as the most preferred roosting tree species, accounting for 16.54% of total observations. The study emphasizes the importance of specific tree attributes, such as height and canopy area, in roost site selection within the urban campus environment. The research contributes to a better understanding of bat roosting ecology and provides insights to guide conservation strategies, recommending that urban planners prioritize the preservation of preferred tree species like *Celtis zenkeri*. Additionally, statistical analyses, including a one‐way ANOVA, confirmed significant preferences for tree heights ranging from 15 to 20 m. This research highlights the need for targeted conservation strategies that consider the specific roosting habits of bats in urban settings, emphasizing the importance of preserving key tree species and habitats within rapidly urbanizing landscapes.

## Graphical abstract

This study investigates the spatial distribution and roosting preferences of Straw‐Colored Fruit Bats ( *Eidolon helvum* ) within the Obafemi Awolowo University campus in Nigeria. Using field surveys and GIS analysis, we identified significant roosting sites predominantly in Celtis zenkeri trees, highlighting the importance of tree height and canopy cover in roost selection. Our findings emphasize the need for targeted conservation strategies to protect critical habitats in urban environments.

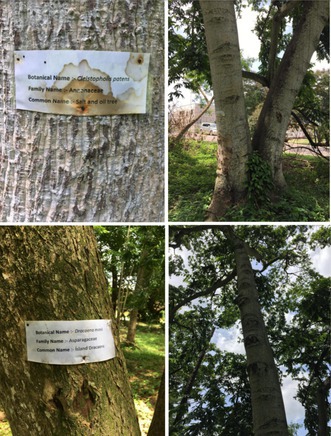

## 1 Introduction

**FIGURE 1:**
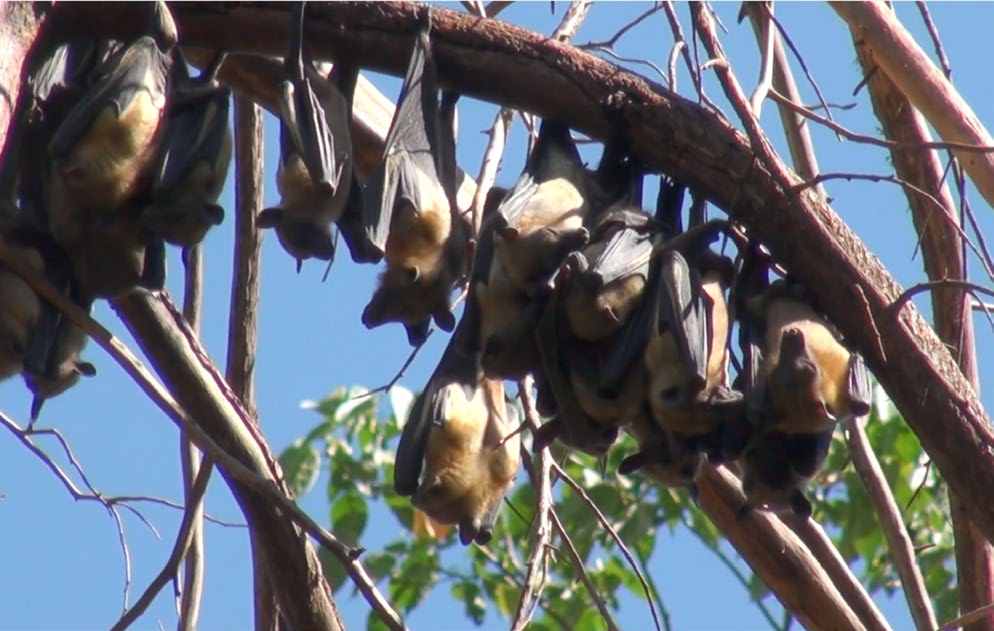
A photograph of the Straw‐Colored Fruit Bat ( *Eidolon helvum* ) roosting in a tree. This image captures the bats in their natural habitat, showcasing their social behavior and communal roosting patterns. The photograph highlights the species' distinctive features, including their size and coloration, which are crucial for identification and understanding their ecological role (*Photo credit: Ndebele M*).

The Straw‐Colored Fruit Bat ( *Eidolon helvum* ) is a significant species in many ecosystems, known for its role in pollination and seed dispersal (Figure 1).

Bats play a crucial role in ecosystem functioning as pollinators, seed dispersers, and insect controllers. They are widely distributed across the globe, with over 1400 species found in various habitats (Ramírez‐Fráncel et al. 2022). One particular species of interest is the Straw‐Colored Fruit Bat ( *Eidolon helvum* ), a large frugivorous bat endemic to sub‐Saharan Africa (Montecino‐Latorre et al. 2022; Detweiler 2023).

The spatial distribution and roosting behavior of bats are influenced by both natural and anthropogenic factors (Arumoogum et al. 2019). Natural factors such as the availability of roosting sites, food resources, and suitable climatic conditions largely determine bat habitat preferences (Suominen et al. 2023). For instance, studies have found that Straw‐Colored Fruit Bats exhibit a strong preference for tall trees. Roost fidelity has also been observed, with individuals often returning to the same roosting sites over consecutive nights or even seasons (Hoff et al. 2024; Diengdoh et al. 2022).

On the other hand, anthropogenic factors, including urbanization, habitat fragmentation, and human‐wildlife conflicts, can pose significant threats to bat populations (Detweiler and Bernard 2023). Urbanization, in particular, can have a detrimental impact on bat populations, as it often leads to the loss and degradation of natural habitats (Alencastre‐Santos et al. 2024). However, urban environments can also provide alternative roosting opportunities, such as buildings, bridges, and even streetlights (Detweiler and Bernard 2023).

Straw‐Colored Fruit Bats are known for their ecological and economic importance. As frugivores, they play a vital role in seed dispersal and forest regeneration (Durand‐Bessart et al. 2023). Additionally, these bats are considered a valuable resource for ecotourism and have cultural significance in some communities (Umar et al. 2024). However, their populations face various threats, including hunting, habitat loss, and the impacts of climate change (Downs et al. 2012).

Despite the growing body of research on urban bat roosting, studies specifically investigating the roosting behavior of Straw‐Colored Fruit Bats in urban environments remain scarce (Tomori and Oluwayelu 2023). In Africa, the Straw‐Colored Fruit Bat is a well‐documented species known for its frugivorous diet and extensive roosting habits. However, studies specifically examining the roosting behavior of Straw‐Colored Fruit Bats in urban settings, particularly in Nigeria, remain scarce. Existing research tends to focus on bats in more rural or less disturbed environments, overlooking the dynamics of urban bat populations (Frick et al. 2020).

Research has shown that Straw‐Colored Fruit Bats exhibit a strong preference for tall trees, which provide optimal roosting and foraging opportunities (Abedi‐Lartey 2016). These bats are known to favor large trees not only for roosting but also for fruiting, as they rely on these resources for sustenance. Additionally, other fruit bat species, such as flying foxes and Rousettus, also demonstrate similar preferences for specific tree types and structural attributes (Singh 2023).

The selection of roosting sites is influenced by various factors, including food availability, competition, and favorable climatic conditions (Jankowiak et al. 2015). Bats tend to thrive in environments that offer a rich diversity of food sources and minimal competition from other species (Salinas‐Ramos et al. 2020). This adaptability is crucial for their survival, especially in urban settings where natural habitats are increasingly fragmented (Delaval et al. 2005).

The main objective of this study is to establish the spatial distribution of available roosts of Straw‐Colored Fruit Bats within the OAU campus and investigate the factors influencing their selection of roosting sites (Mickleburgh et al. 2002). This study hypothesizes that the spatial distribution of Straw‐Colored Fruit Bat roosts within the Obafemi Awolowo University campus is significantly influenced by both environmental predictors (e.g., tree height and species) and climatic conditions (e.g., temperature and humidity).

Understanding the roosting ecology of this species in an urban setting, such as the OAU campus, can provide valuable insights into their adaptability and inform conservation strategies (Becker 2023). This knowledge can contribute to urban planning and wildlife management efforts, aiming to balance the needs of bats with human activities (Gaultier 2023).

## 2 Materials and Methods

### 2.1 Study Area

**FIGURE 2:**
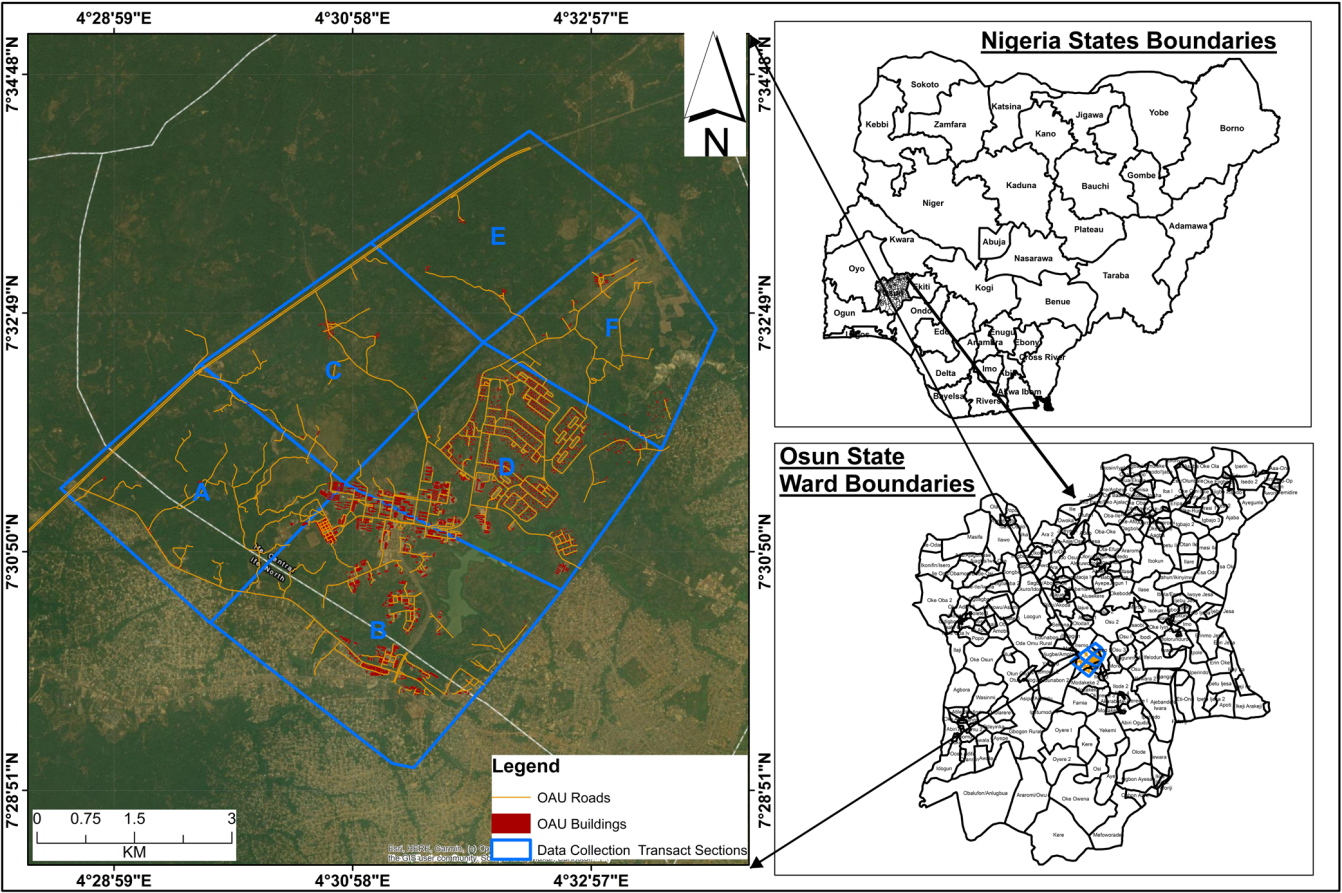
Map showing Obafemi Awolowo University campus and study sites within districts of Nigeria.

The study was conducted within the OAU campus, located in the city of Ile‐Ife in Osun State, Nigeria (Ifeoluwa 2023). The campus spans approximately 11 km^2^ and features a mix of habitats, including woodlands and gardens, providing diverse ecological niches for various fauna. Notable native plant species include *Khaya senegalensis* (African mahogany), *Terminalia catappa* (tropical almond), and *Ficus sycomorus* (sycamore fig). The campus is also home to a variety of fauna, including bird species such as the common weaver ( *Ploceus cucullatus* ) and the olive pigeon ( *Columba arquatrix* ), small mammals like the African giant rat ( *Cricetomys gambianus* ) and bushy‐tailed mongoose ( *Bdeogale crassicauda* ), as well as various invertebrates, including butterflies from the genus *Papilio* and common garden snails (*Cornu aspersum*). The campus is situated in the southwestern region of the country, bounded by the coordinates 7°28′ N, 4°33′ E in the northwest and 7°32′ N, 4°37′ E in the southeast (Figure 2) (Okeowo 2021). The campus landscape is characterized by a mix of habitats, including woodlands, gardens, and open spaces. The woodlands are composed of a variety of native and introduced tree species, providing important ecological niches for diverse fauna (Omokunle et al. 2024). The well‐maintained campus gardens offer pockets of greenery that attract both human visitors and wildlife. Interspersed throughout the campus are open areas that facilitate interactions between the academic community and the local wildlife (Adewoyin 2023).

As of 2023, the OAU campus has a student population of over 35,000 and a faculty and staff population of approximately 5000 (Igbo 2023). In addition to the human population, the campus is home to a diverse array of flora and fauna, including various bird species, small mammals, and a range of invertebrates. The unique blend of academic activities, green spaces, and diverse habitats within the OAU campus provides an ideal setting for investigating the roosting behavior and habitat preferences of the Straw‐Colored Fruit Bat ( *Eidolon helvum* ) (Adesoji et al. 2024), a large frugivorous bat species endemic to sub‐Saharan Africa.

**FIGURE 3:**
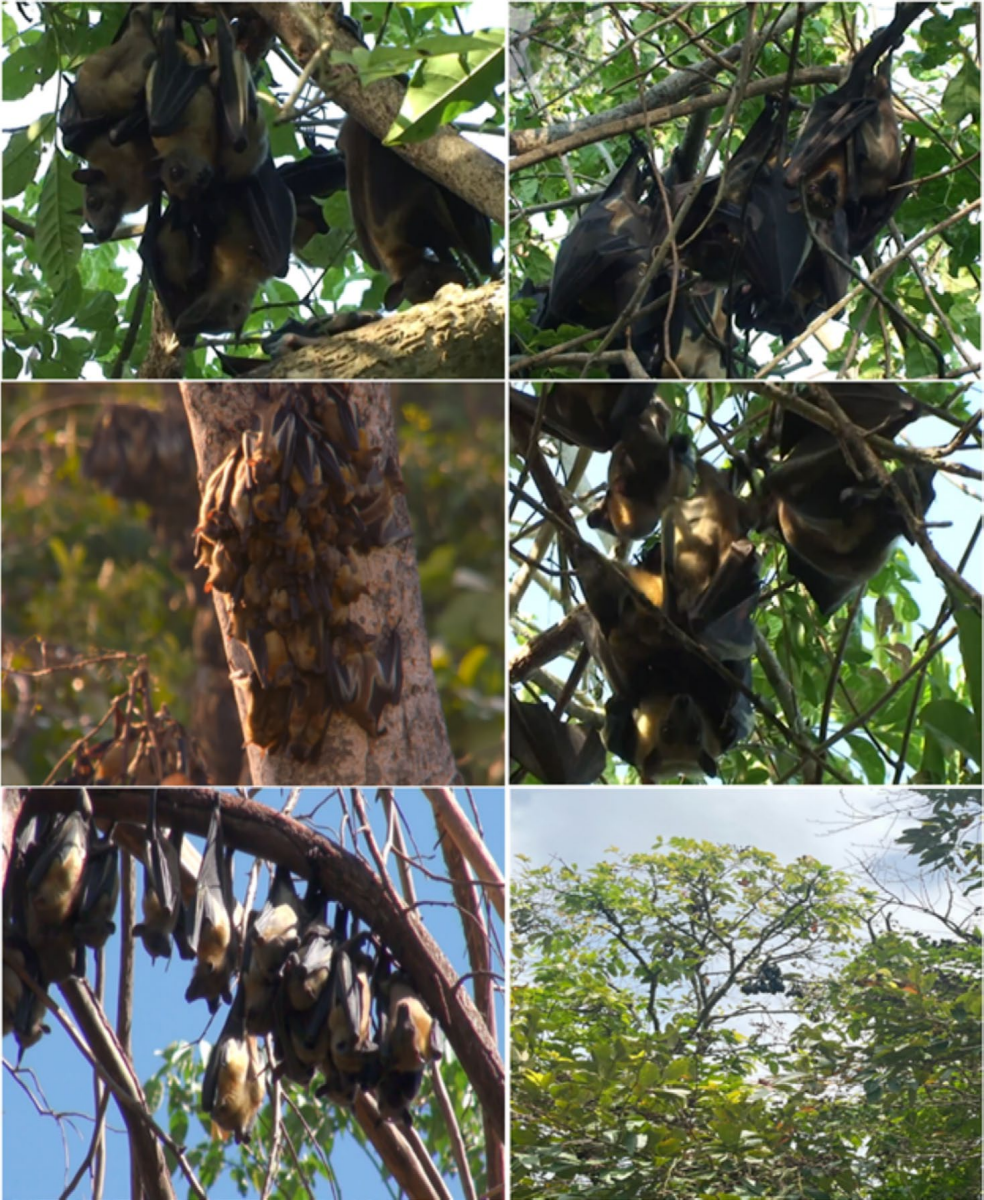
Photo grid of the Straw‐Colored Fruit Bat ( *Eidolon helvum* ) in various roosting sites across the Obafemi Awolowo University campus. This collection of images, captured by the authors during data collection, highlights the diverse habitats and key features of the species in its natural environment, emphasizing its roosting behavior and preferences (*Photo credit: Ndebele M, AbdulAfeez S*).

As shown in Figure 3, the Straw‐Colored Fruit Bat ( *Eidolon helvum* ) exhibits distinctive features that are critical to understanding its roosting behavior. This large frugivorous bat is characterized by its striking golden‐brown fur and elongated wings, which enable agile flight and efficient foraging in various habitats. Its large eyes are adapted for low‐light conditions, allowing it to navigate and locate food sources effectively during dusk and dawn. The bat's preference for roosting in tall trees, particularly *Celtis zenkeri*, is reflected in its physical adaptations, including strong claws that facilitate gripping onto branches. Understanding these features not only provides insights into the bat's ecological role as a seed disperser but also highlights the importance of suitable roosting sites in urban environments.

### 2.2 Data Collection

The study employed a multifaceted approach to explore the spatial distribution of accessible roosts utilized by the Straw‐Colored Fruit Bats within the confines of OAU campus. This endeavor entailed a harmonious blend of field surveys, data compilation via Kobo Collect, and geospatial analysis. The overarching methodology was meticulously designed to procure insights into roosting behaviors, the attributes of roosts, and the role of environmental variables in shaping roost distribution. Field surveys were conducted from May 15, 2023, to May 29, 2023, encompassing a duration of 2 weeks. These surveys were primarily undertaken during the late morning hours, which coincided with the period when bats were roosting and inactive. This timing facilitated the identification of roosting sites, thereby maximizing the accuracy of observations without the interference of bats in flight.

The study employed a stratified sampling approach, with 6 transects established across different sections of the OAU campus. The campus was divided into 6 distinct zones, labeled A through F, each measuring approximately 2.5–3 km by 2.5–3 km, depending on accessibility and terrain. Within each zone, parallel transects were laid out, spaced approximately 100 m apart. The researchers systematically walked along these transects, carefully examining trees and documenting the presence of Straw‐Colored Fruit Bat roosts. This approach ensured comprehensive coverage of the study area and allowed for the spatial distribution of the roosts to be accurately mapped. A total of 156 distinct roost sites were identified and recorded. The methodology involved the identification of tree species, for which scientific names were obtained from tags placed by the university's biological sciences department. Additionally, environmental variables were assessed for each identified roosting tree, including estimated tree height, basal area, and canopy area; tree height was measured using a laser rangefinder.

**FIGURE 4:**
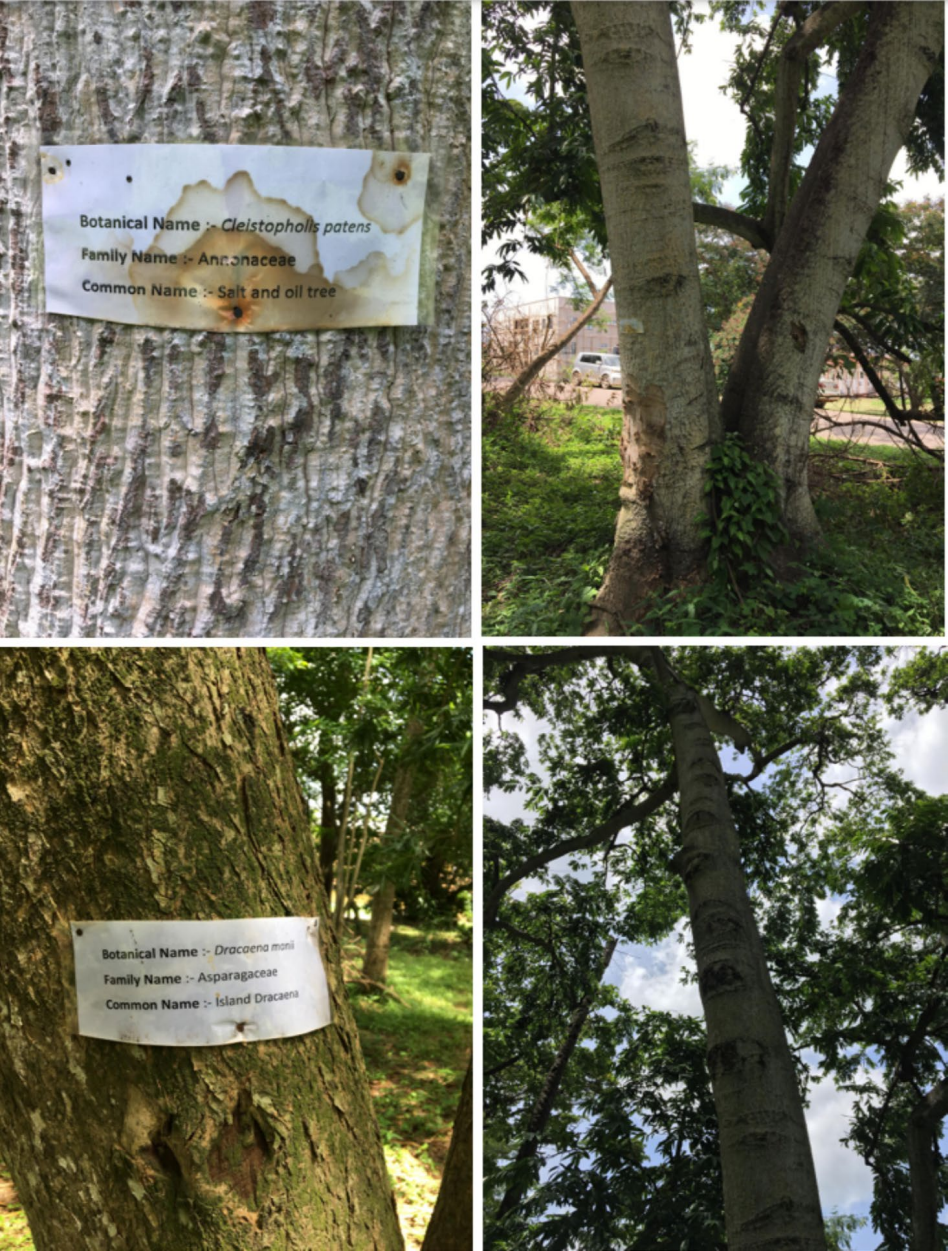
Photo grid of examples of tree species name tags used for identification. These images, taken by the authors during data collection, illustrate the labeling of various tree species encountered in the study area (*Photo credit: Ndebele M, AbdulAfeez S*).

The study incorporated comprehensive field surveys, predominantly conducted during the late afternoon hours—a period coinciding with peak bat activity. During these surveys, researchers traversed various segments of the OAU campus, systematically observing and documenting trees housing bats. The scientific names of the tree species were obtained from tags placed on the tree trunks by the university's biological sciences department, which has labeled almost every tree species on the campus (Figure 4). This direct, on‐site approach enabled the collection of real‐time data regarding roosting patterns and the diverse tree species preferred by the bats.

### 2.3 Data Collection Using Kobo Collect

The Kobo Collect mobile application served as a robust tool for the systematic acquisition of relevant data on the Straw‐Colored Fruit Bat roosting sites across the OAU campus. For each identified bat roosting tree, the following essential parameters were meticulously recorded: estimated tree height, measured using a laser rangefinder; tree species identification, performed through visual inspection and consultation of plant taxonomy references; basal area at breast height, calculated from trunk circumference measurements; estimated canopy area, visually assessed by the researcher; and the presence and identity of any Straw‐Colored Fruit Bats observed in the vicinity. In conjunction with these data points, the GPS coordinates of each identified roosting tree were logged using the built‐in GPS functionality of the Kobo Collect application (Ndebele and Mazhindu 2023). This comprehensive data compilation was supplemented by capturing photographic documentation of the roosting trees and their immediate surroundings, enriching the dataset for subsequent analysis. Using Kobo Collect ensured a standardized and systematic approach to data collection, facilitating efficient and accurate recording of the necessary parameters for studying the roosting ecology of Straw‐Colored Fruit Bats on the OAU campus. Its user‐friendly interface and offline capabilities made it particularly suitable for fieldwork, allowing us to focus on gathering high‐quality data in a timely manner (Mekuriaw et al. 2024).

### 2.4 Mapping Roosting Sites Distribution

**FIGURE 5:**
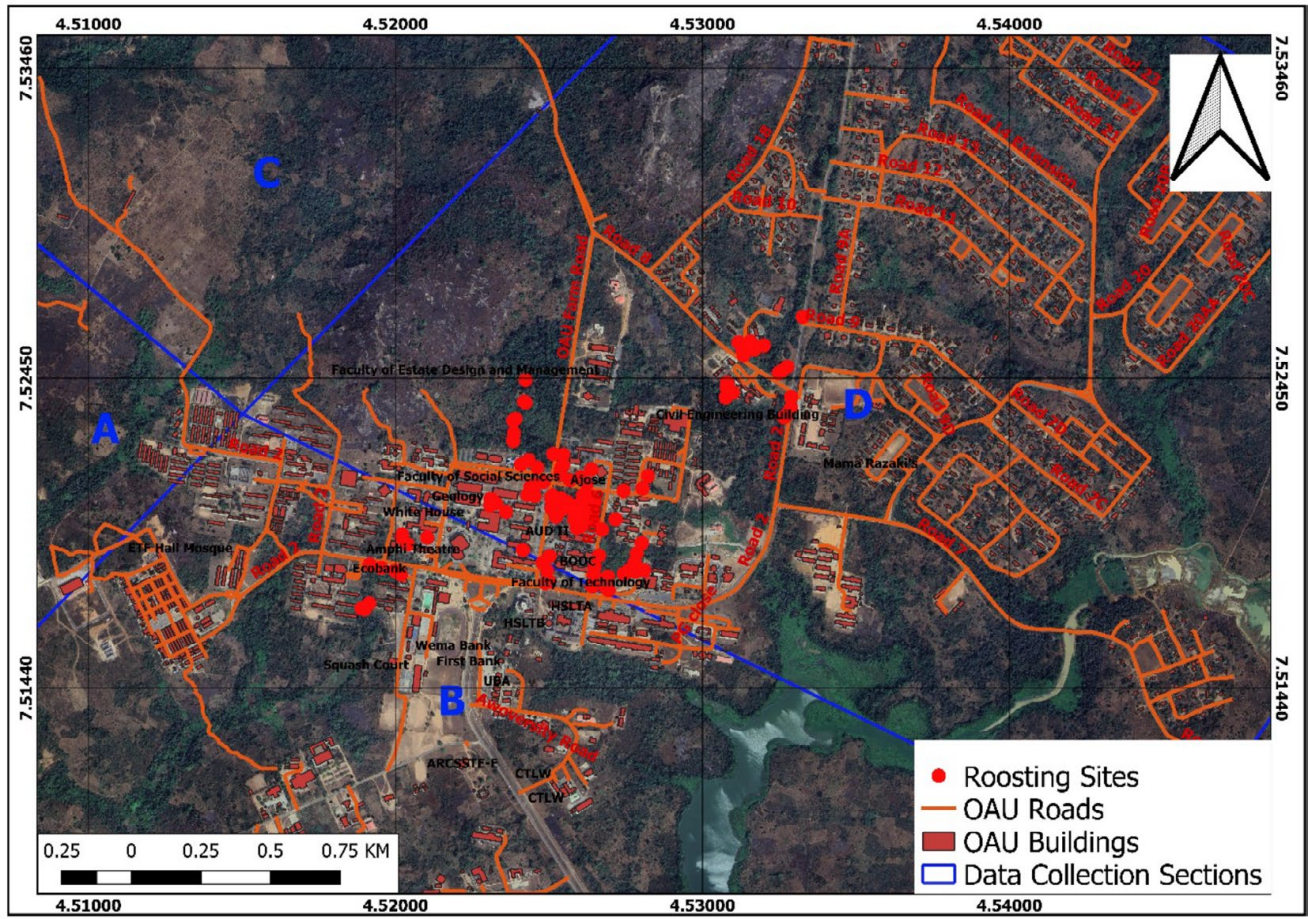
Spatial distribution of roosting sites.

The GPS coordinates of the roosting trees were imported into Quantum GIS to visualize the spatial distribution of bat roosting places within the OAU campus. The GPS points were plotted on a map, allowing for a comprehensive view of the distribution patterns across the study (Figure 5).

### 2.5 Data Analysis

The data collected during the field surveys were analyzed using both statistical and geospatial methods to evaluate the preferences of Straw‐Colored Fruit Bats for roosting sites. Geospatial analysis involved the use of Geographic Information Systems (GIS) to map the distribution of identified roosting sites across the OAU campus. This mapping allowed for the visualization of spatial patterns and the identification of hotspots where bats were most frequently observed. To reduce uncertainty in the analysis, factors such as tree species, height, and environmental conditions were considered. Statistical techniques, including one‐way ANOVA, were employed to assess differences in tree heights between roosting and non‐roosting sites. This analysis provided insights into the specific height preferences of the bats, revealing significant trends in their roosting behavior.

To evaluate the accuracy of the geospatial analysis, we conducted a validation process involving ground‐truthing of a subset of the mapped roosting sites. This involved revisiting selected locations to confirm the presence of bat roosts and ensuring the reliability of the data collected. Additionally, the accuracy of the GIS mapping techniques was assessed by comparing the modeled distribution of roosts with actual observations, allowing for adjustments to improve the precision of the analysis. Advanced analysis of associated tree height was performed using regression techniques to explore the relationship between tree height and the likelihood of roosting. This involved fitting a logistic regression model to predict the probability of a tree being used as a roost based on its height and other environmental variables. The results of this analysis provided a deeper understanding of the ecological factors influencing roost selection by the Straw‐Colored Fruit Bats.

### 2.6 Statistical Analysis

To analyze roosting preferences, statistical tests were conducted using R software. A one‐way ANOVA was performed to determine differences in tree height between roosting and non‐roosting sites, with the null hypothesis (H0) stating that there is no significant difference in tree heights between the two site types, while the alternative hypothesis (H1) posits that a significant difference exists. The results of the ANOVA revealed a significant difference in tree heights, with an *F*‐value of 1.48 and a *p*‐value of 0.001. Post hoc comparisons using Tukey's HSD test indicated that roosting sites had significantly greater tree heights than non‐roosting sites (*p* < 0.01). Therefore, the null hypothesis was rejected, supporting the conclusion that tree height significantly influences roosting site selection.

### 2.7 Geospatial Analysis

The gathered data were subjected to rigorous geospatial analysis using Quantum GIS 3.30.2, a robust geographic information system (GIS) software (Chakuya et al. 2024). The primary focus of this analysis was to unravel the spatial distribution of Straw‐Colored Fruit Bat roosts within the intricate fabric of the OAU campus. This encompassed the mapping of roosting sites, discernment of hotspot clusters, identification of spatial patterns, and exploration of preferences for particular tree species as roosting sites of these bats.

### 2.8 Kernel Density Estimation

Kernel density estimation (KDE) was used to investigate the spatial distribution and potential roosting hotspots of the Straw‐Colored Fruit Bats within the OAU campus. The KDE analysis was conducted based on the roosting site data collected during the comprehensive field surveys (Medinas et al. 2021).

The KDE was performed using Gaussian kernel functions, which defined the influence zones around each recorded roosting observation. This approach allowed the researchers to visualize the density of roosting sites across the study area, highlighting the locations with the highest concentration of bat roosts. By integrating the KDE results with the detailed field observations, the study was able to provide valuable insights into the roost site selection preferences of the Straw‐Colored Fruit Bats. The KDE analysis revealed the spatial clustering of roosting sites, indicating that the bats exhibited a non‐random distribution pattern and preferentially selected certain areas within the OAU campus for roosting. This information is crucial for understanding the factors influencing roost site selection, such as the availability of suitable tree species, vegetation structure, and proximity to foraging resources (Seal et al. 2022). To further strengthen the analysis of roost site selection, the researchers plan to investigate the specific characteristics of the preferred roosting trees and the surrounding habitat features. This will involve a more detailed examination of the tree species, canopy cover, proximity to water sources, and other environmental variables that may influence the bats' roosting preferences. By combining the KDE results with a more comprehensive analysis of roost site attributes, the study aims to provide a deeper understanding of the Straw‐Colored Fruit Bats' roosting ecology within the OAU campus.

## 3 Results

The analysis of the 156 roosting observations collected during the study revealed distinct spatial patterns in the distribution of roosting sites across the OAU campus. The observations were concentrated in specific sections, with the majority (130 observations, 83%) located in Section D, followed by 27 observations (17%) in Section B. The remaining sections, C, F, A, and E, had no recorded roosting observations. In terms of estimated tree height preferences, the most favored range fell within 15–20 m, with the tallest observed tree nearly reaching 30 m in height. On the lower end, the shortest trees measured between 5 and 8 m, indicating that Straw‐Colored Fruit Bats exhibited a preference for trees of substantial height for their roosting activities. The tree height measurements were obtained using a laser rangefinder during the field surveys conducted across the different sections of the study area.

**FIGURE 6:**
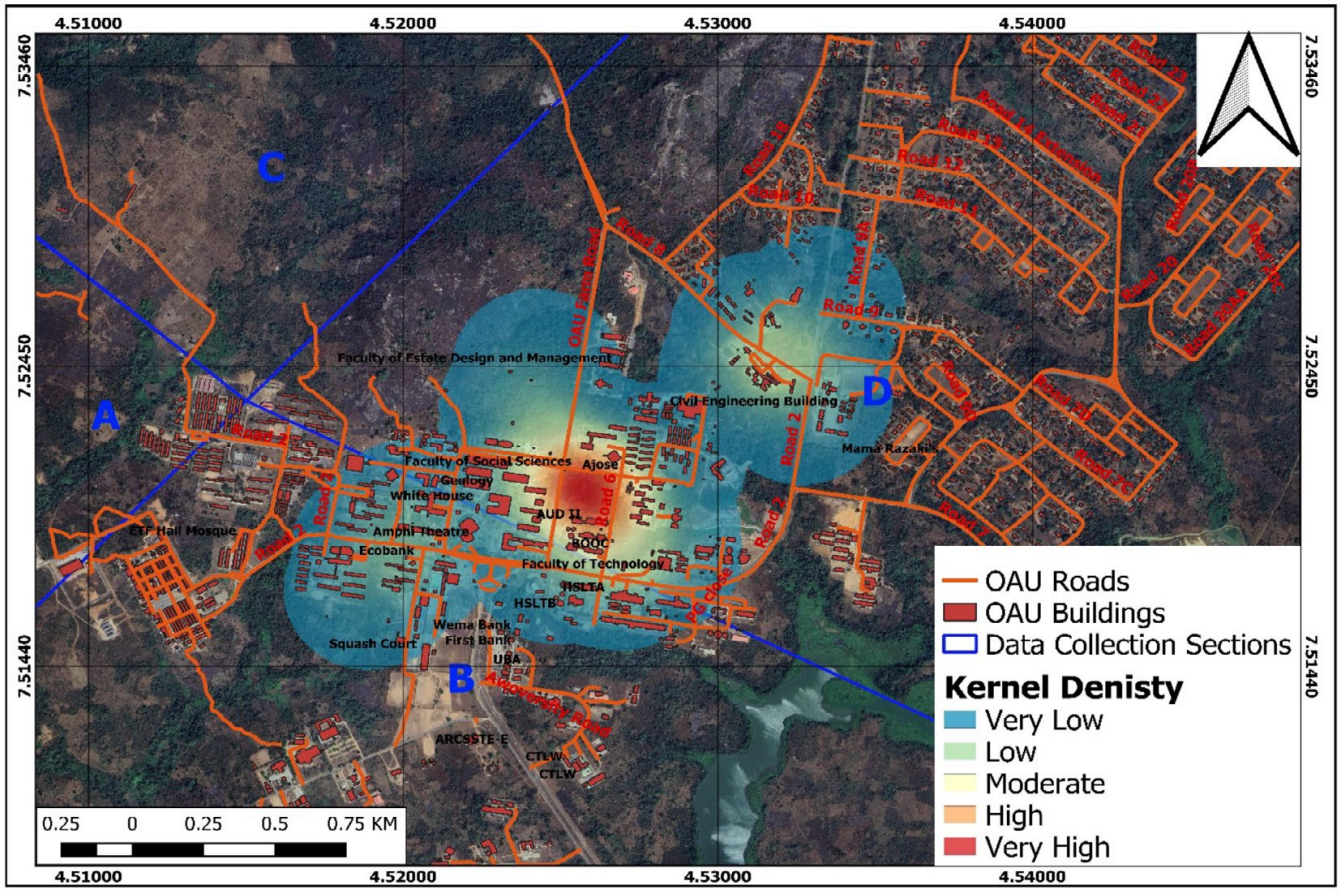
Kernel density estimation for the potential roosting sites.

The sections with the highest number of roosting sites were Sections D and B, with Section D having the majority of observations (130 observations, 83%) and Section B having the remaining observations (27 observations, 17%). These sections were characterized by the presence of tall trees with large canopy cover, as well as areas with high levels of human activity. In contrast, the remaining sections, A, C, E, and F, had no observed roosting sites. The spatial clustering of the roosting sites was further confirmed by the KDE analysis, which identified two prominent hotspots of roosting activity (Figure 6). The first hotspot was centered around the Biological Gardens next to the Zoology Department, while the second hotspot was found around the Conference Center, both of which were located within Section D. These areas provided the ideal combination of roosting habitat features, including the presence of tall buildings, moderate light intensities, and suitable roosting structures such as crevices and cavities.

The findings suggest that the Straw‐Colored Fruit Bats inhabiting the OAU campus prefer to roost in areas with a high density of tall trees and moderate levels of human activity. The proximity of the roosting hotspots to the Faculty of Science and the Department of Biological Sciences indicates that these bats may be taking advantage of the abundant resources and potential food sources available in these academic and research‐focused areas. These insights into the roosting ecology of the OAU bats provide a valuable foundation for developing targeted conservation strategies. The identified hotspot areas should be prioritized for habitat protection and management, ensuring the preservation of the critical roosting sites for these ecologically important species. Further research is recommended to investigate seasonal variations in roosting patterns, as well as the potential impacts of land use changes and building modifications on the bats' roosting preferences.

### 3.1 Analysis of Tree Species Preferences

During field surveys, we conducted an in‐depth analysis of the tree species data collected to discern the specific tree preferences of the Straw‐Colored Fruit Bats as roosting sites. We meticulously examined the frequency and distribution of diverse tree species that were utilized by the bats. This analysis was conducted with the intention of identifying the tree species that exhibited the highest favorability as roosting locations within the OAU campus ecosystem.

*Celtis zenkeri* emerged as the most preferred tree species among the Straw‐Colored Fruit Bats, accounting for 16.54% of the total observations. Following closely was *Delonix regia* , representing 8.27% of observations, and *Moraceae* at 7.52%. Other tree species received preferences below the 5% mark, with the majority registering a preference of 0.75%. This pronounced preference for *Celtis zenkeri* aligns with its suitability as a roosting site within the campus habitat (Table 1).

**TABLE 1:** Tree species observed as roosting sites.

Tree species	Count	%
*Celtis zenkeri*	22	16.54
*Delonix regia*	11	8.27
*Moraceae*	10	7.52
*Ebanaceum*	7	5.36
*Milicia excelsa*	6	4.51
*Enterolobium cyclocarpum*	5	3.76
*Cedrela toona*	4	3.01
*Neem tree*	4	3.01
*Azadirachata indica*	3	2.26
*Gemelina arborea*	3	2.26
*Agrilus prionurus*	2	1.50
*Anarcardium excelsum*	2	1.50
*Cedrus atlantica* ‘*Glauca*’	2	1.50
*Focus callosa*	2	1.50
*Kapok tree*	2	1.50
*Magnifera indica*	2	1.50
*Palm tree*	2	1.50
*Sterculia tragacantha*	2	1.50
*Thuja plicata*	2	1.50
*Thyrsodium spruceanum*	2	1.50
*Tieghemella heckelii*	2	1.50
*Diospyros bipindensis*	1	0.75
*Acacia mangium*	1	0.75
*Adina cordifolia*	1	0.75
*Alangium ebanaceum*	1	0.75
*Albizia zygea*	1	0.75
*Aphanamixis polystachya*	1	0.75
*Banyan pohon*	1	0.75
*Bigleeaf magnolia*	1	0.75
*Blighia sapida*	1	0.75
*Calamus vitiensis*	1	0.75
*Canarium schweinfurthii*	1	0.75
*Ceba pentandra*	1	0.75
*Cebu cinnamon*	1	0.75
*Cleistopholis patens*	1	0.75
*Dalbergia oliveri*	1	0.75
*Demonic regia*	1	0.75
*Diospyros bipindensis*	1	0.75
*Dracaena manii*	1	0.75
*Erythrina stricta*	1	0.75
*Ficus* sp.	1	0.75
*Firmiana simplex*	1	0.75
*Funtumia elastica*	1	0.75
*Khaya grabdifoliola*	1	0.75
*Millettia laurentii*	1	0.75
*Octlolobus Grandis*	1	0.75
*Oldfieldia africana*	1	0.75
*Pentadesma butyracea*	1	0.75
*Pisonia grandis*	1	0.75
*Pletophorum pterocarpum*	1	0.75
*Quercus robur*	1	0.75
*Riccinodondron*	1	0.75
*Syzygium branderhorstii*	1	0.75
*Tessmannia Aficana*	1	0.75
*Tetrapleura traptera*	1	0.75
*Triplochiton scleroxylon*	1	0.75
*Zygia latifiola*	1	0.75

In terms of estimated tree height preferences, the most favored range fell within 15–20 m, with the tallest observed tree nearly reaching 30 m in height. On the lower end, the shortest trees measured between 5 and 8 m. A one‐way ANOVA test revealed a statistically significant difference in the tree heights of bat roosting sites compared to non‐roost sites (*F*(1, 48) = 12.78, *p* < 0.001), indicating that Straw‐Colored Fruit Bats exhibited a strong preference for taller trees for their roosting activities.

The estimated canopy area also played a role in roost selection. Larger canopy areas, ranging from 314.159 to 706.858 square meters, seemed to hold a particular allure for the bats. In contrast, trees with smaller canopy areas, spanning from 12.566 to 50.265 square meters, were also used as roosts but appeared to be favored to a lesser extent. To further investigate the influence of canopy cover on roost site selection, the study area was divided into 50 × 50‐m grid blocks, and the canopy cover within each block was quantified using remote sensing data. A Pearson correlation analysis revealed a significant positive correlation between the canopy cover per block and the number of bat roosts observed (*r* = 0.67, *p* < 0.01), suggesting that the availability of dense canopy cover was a key factor in the bats' roost site selection. Additionally, the study found that the bats exhibited a clear preference for trees with specific bark characteristics, favoring those with rough, fissured bark that provided ample crevices and cavities for roosting. This preference for trees with textured bark was observed across all the identified roosting sites, further underscoring the bats' selective nature when it comes to their choice of roosting locations.

## 4 Discussion

These insights into the preferences of Straw‐Colored Fruit Bats for specific tree species, as well as their height and canopy characteristics, contribute to the understanding of the intricate ecological dynamics governing roost selection (Kerr, n.d.) within the OAU campus and other areas with similar conditions. Such preferences underscore the complex interactions between bats and their habitat, shedding light on the delicate balance that shapes the spatial distribution of these creatures within their urban environment (Stein 2023). This study provides critical insights into the roosting behavior of Straw‐Colored Fruit Bats within the Obafemi Awolowo University campus, highlighting their strong preference for Celtis zenkeri trees adjacent to built infrastructure. These findings not only enhance our understanding of the species' ecological requirements but also underscore the importance of urban habitats in supporting wildlife.

### 4.1 Ecological Implications

The preference for *Celtis zenkeri* suggests that this species plays a vital role in the local ecosystem, providing essential roosting sites that facilitate foraging and reproductive success. The structural attributes of *C. zenkeri* , including its height and canopy cover, likely contribute to its suitability as a roosting tree. The presence of these trees in close proximity to urban structures may offer advantages such as reduced predation risk and increased thermal stability, which are crucial for the bats' survival in urbanized environments (Jung and Threlfall 2016).

These findings align with existing literature indicating that urban environments can offer alternative habitats for bats, despite the potential threats posed by habitat fragmentation and human activity. The ability of Straw‐Colored Fruit Bats to adapt to urban landscapes is indicative of their ecological resilience, yet it also raises concerns regarding their long‐term survival in the face of ongoing urbanization (Suárez Paladines 2022).

### 4.2 Contextualizing Bats in Africa

Bats play a critical role in maintaining ecological balance across various African ecosystems. As pollinators and seed dispersers, they contribute significantly to forest regeneration and biodiversity (Aziz et al. 2021). Despite their ecological importance, bats are often overlooked in conservation discussions, particularly in urban settings where habitat loss poses substantial threats. Studies have shown that urbanization can alter bat distribution and behavior, necessitating targeted conservation strategies (Jung and Threlfall 2016).

### 4.3 Implications for Conservation

The identified roosting hotspots within the university campus present important implications for conservation management. By prioritizing the preservation of *C. zenkeri* and other critical tree species, urban planners and conservationists can create green corridors that support bat populations and enhance biodiversity. Efforts to maintain and expand these habitats can be integrated into campus management plans, ensuring that the ecological needs of bats are considered alongside human activities. Moreover, the study highlights the need for ongoing monitoring of bat populations in urban settings. The establishment of long‐term research initiatives could provide valuable data on how roosting preferences may change over time, particularly in response to environmental shifts, urban development, and climate change. Understanding these dynamics will be essential for developing effective conservation strategies tailored to the unique challenges faced by urban bat populations (Shwartz et al. 2014).

### 4.4 Limitations and Future Research Directions

While this study has provided significant insights, it is important to acknowledge its limitations. The confinement of the research to a single campus restricts the generalizability of the findings. Future studies should include comparative analyses across various urban and rural settings to isolate true species‐level preferences from site‐specific factors. In particular, a comparison of different roosting sites, such as forests and caves outside the university, would enhance the understanding of habitat selection.

Incorporating tree availability mapping will further strengthen the analysis, allowing for a clearer understanding of whether bats are actively selecting *C. zenkeri* over other available tree species.

Additionally, the lack of seasonal coverage presents another limitation. Roost site use may vary with changing environmental conditions, and future research should encompass year‐round sampling to capture these dynamics. A more comprehensive approach that includes seasonal variations in roosting behavior, as well as the potential impacts of food resource availability, will provide a deeper understanding of the ecological requirements of Straw‐Colored Fruit Bats.

## 5 Conclusion

This comprehensive study on the roosting behavior and spatial distribution of the Straw‐Colored Fruit Bat within the OAU campus represents a significant contribution to urban bat ecology. Employing extensive field surveys and rigorous quantitative analyses, the research has shed invaluable light on the bats' preferences for specific tree species, heights, and canopy characteristics, underscoring the complex interplay between these mammals and their rapidly transforming urban environment. The mapped distribution of roosting sites and identified hotspot clusters provide a powerful foundation for targeted conservation initiatives, allowing stakeholders to focus efforts on key areas crucial to the bats' survival. As urbanization continues to reshape ecosystems, this study emphasizes the critical role that universities and institutions can play in balancing development with ecological preservation. The findings underscore the importance of maintaining diverse habitats within urban areas, ensuring the needs of both wildlife and human communities are carefully considered. In conclusion, this research serves as a guidepost for sustainable urban planning that prioritizes the preservation of critical wildlife habitats and recommends that city planners and policymakers incorporate these insights to inform future development strategies and conservation efforts for the Straw‐Colored Fruit Bat and other urban‐adapted species.

## Author Contributions

**Manuel Ndebele:** conceptualization (lead), data curation (equal), formal analysis (lead), methodology (lead), project administration (lead), resources (equal), software (lead), supervision (lead), validation (lead), visualization (equal), writing – original draft (lead), writing – review and editing (equal). **Mary Ebun Ajibola:** conceptualization (equal), data curation (equal), formal analysis (equal), methodology (equal), project administration (equal), supervision (equal), writing – original draft (equal), writing – review and editing (equal). **Jerimiah Chakuya:** conceptualization (supporting), data curation (supporting), formal analysis (supporting), investigation (equal), methodology (equal), project administration (supporting), resources (equal), software (supporting), supervision (supporting), validation (equal), visualization (equal), writing – original draft (supporting), writing – review and editing (equal). **Suara AbdulAfeez:** conceptualization (equal), data curation (equal), formal analysis (equal), investigation (equal), methodology (equal), project administration (lead), resources (equal), software (equal), supervision (equal), validation (equal), visualization (equal), writing – original draft (equal), writing – review and editing (equal). **Pamilerin Ajumobi:** data curation (equal), formal analysis (equal), investigation (equal), project administration (equal), resources (equal), validation (equal), visualization (equal), writing – original draft (equal), writing – review and editing (equal). **Faith Adeniran:** data curation (equal), investigation (equal), methodology (equal), project administration (equal), resources (equal), software (equal), validation (equal), writing – original draft (equal), writing – review and editing (equal). **Busayo Aina:** conceptualization (equal), data curation (equal), formal analysis (equal), investigation (equal), methodology (equal), resources (equal), visualization (equal), writing – review and editing (equal). **Toheeb Oladapo:** conceptualization (equal), data curation (equal), investigation (equal), software (equal).

## Conflicts of Interest

The authors declare no conflicts of interest.

## Data Availability

The research data related to this study, including the mapped roost distribution, hotspot identification, and tree species preferences for Straw‐Colored Fruit Bats (*Eidolon helvum*), can be accessed at the following link: Roosting sites for Straw‐Colored Fruit Bats (https://figshare.com/s/e2bd4a1f5971edc55960?file=53199971).
